# Pupillary Responses to Illusions of Brightness in Autism Spectrum Disorder

**DOI:** 10.1177/2041669518771716

**Published:** 2018-05-17

**Authors:** Bruno Laeng, Fredrik Svartdal Færevaag, Stine Tanggaard, Stephen von Tetzchner

**Affiliations:** Department of Psychology, University of Oslo, Norway

**Keywords:** visual illusion, brightness illusion, pupillary response, pupillometry, autism spectrum disorder

## Abstract

Previous studies indicate that individuals with autism spectrum disorder (ASD) do not experience optical illusions in the same manner as individuals with typical development. This study uses pupillary responses as an objective measure of perception of visual illusions, with the hypothesis that adults with ASD will show weaker pupillary constrictions to the illusions than adults without ASD. An eye-tracker was used to investigate the spontaneous pupillary changes to brightness illusions in adults diagnosed with ASD (*N* = 11) and in a control group (*N* = 24). Contrary to the hypothesis, the ASD group showed similar pupillary constrictions to the illusory bright stimuli as the control group. Therefore, this study does not support the idea that individuals with ASD have a veridical perception of these types of illusions and instead suggest that atypical perception of illusions does not constitute a universal characteristic of aspect of high-functioning individuals with ASD.

It is a general observation that many individuals diagnosed with autism spectrum disorder (ASD; American Psychiatric Association, 2013) have atypical visual perception (Behrmann, Thomas, & Humphreys, 2006; Hadad, Goldstein, & Russo, 2017; Hermelin & O’Connor, 1970; Simmons et al., 2009). Some studies report that individuals with ASD do not experience optical illusions in the same way as individuals with typically development and that, in fact, their perceptions are more realistic or closer to the physical input. For example, Happé (1996) found that individuals with ASD tended to show a lower susceptibility to visual illusions like the Ebbinghaus (aka Titchener) illusion. The two central, black, circles were reported to be of the same size, as they actually are, instead of smaller or larger when surrounded by larger or smaller circles, respectively, as it is in the typical (illusory) perception.

These findings have led to attempts to describe and explain visual processing in individuals with ASD, mainly for two reasons: (a) atypical perceptual processing may reveal a key aspect of ASD that seems different from the main diagnostic features of the disorder (Simmons et al., 2009) and (b) such atypical perception suggests the possibility that the disruption of some fundamental mechanism of perception and learning may relate to a variety of difficulties experienced by individuals with ASD, including those expressed in social communication and behavior (Dakin & Frith, 2005; Hadad et al., 2017).

There are several novel theoretical accounts of atypical perception in people with ASD (Behrmann et al., 2006; Samson, Mottron, Soulieres, & Zeffiro, 2012). Apparently, they all take their starting point from the observation that perception in people with ASD is characterized by a greater reliance than normal on the sensory input, especially in ambiguous situations (Lawson, Rees, & Friston, 2014). These accounts propose a weaker influence of top-down expectations on perception in people with ASD. Normally, prior experiences with sensory information, either at the individual or species level, would shape “perceptual hypotheses” (or “unconscious inferences”; Gregory, 1980; Helmholtz, 1860) that assist the interpretation of incoming information, especially when ambiguous or unexpected (Evers et al., 2014; Hohwy, 2013).

In the visual domain, optical illusions would constitute a paradigmatic example of the operation of top-down factors. That is, the perceptual experience goes beyond the information given and reflects an inference of what is either likely to be present (e.g., a specific three-dimensional shape or scene given only two-dimensional cues) or what is about to take place in the immediately near future (as in the flash-lag illusion or brightness illusions; Changizi, 2009; Hohwy, Paton, & Palmer, 2016). As put by Palmer, Seth, and Hohwy (2015): “What we are perceptually aware of, at any given moment, is the state of the world that is calculated as being most likely to be causing the sensory input that our brain receives” and perception “[…] specifies what sensory input would be received if a certain set of causes existed in the world” (p. 377). Hence, people with ASD may process visual information not only in a different way than other people but, more specifically, according to a cognitive style that can be described as resulting in a more “veridical” perception (as suggested within a Bayesian perspective of vision; see Mottron et al., 2013; Pellicano & Burr, 2012). Specifically, individuals with ASD may attend and perceive the sensory world in a manner that gives them (as a group) “a privileged access to parts and details” (Frith, 1989; Frith & Happé, 1994).

Research on optical illusions can provide a rather privileged window into what “prior hypotheses” the visual system uses when attempting to make sense of ambiguous stimuli. In a previous study from our laboratory on optical illusions of brightness, Laeng and Endestad (2012) showed that fast physiological adjustments of the size of the eye pupil occur when healthy individuals viewed the brightness illusion (illustrated in Figure 1, left). Such a physiological response seems consistent with the interpretation of a scene on the basis of the natural statistics of the world, given that the convergent arrangement of the gradually lighter ends of the elements of the pattern is consistent with a perceptual inference of a bright source of light, at the center of the figure, as it may occur in nature when looking directly at the sun through a canopy of leaves. Intuitively, the experience of an individual (or perhaps of the species) in similar situations has been associated with a particular physical state that could likely lead to “glare” and therefore a “dazzling” effect of strong light on the eyes, a situation that could temporarily incapacitate vision. Hence, a reduction in the size of the pupil would seem to represent a very adaptive response of the visual system to a probable situation, although according to the actual stimulation there is really no difference between the spectral returns of the internal region of the figure and of its surrounding. Thus, visual illusions of this kind can be used to gauge the extent in which (groups of) individuals differ in their use of prior knowledge about the visual world, so that accuracy in perception or “closeness to physical reality” may be either enhanced or reduced (Pellicano & Burr, 2012). Moreover, physiological measures of illusion susceptibility may be particularly valuable for understanding the relationship between autism and illusory perception, since such variables may be less influenced by decisional strategies. Finally, we note the fact that pupil size is outside voluntary control, differently from eye movements or blinking (Laeng, Sirois, & Gredebäck, 2012), and that this measure therefore could provide a unique perspective into the “nonconscious inferences” of the perceptual system.

**Figure 1.**
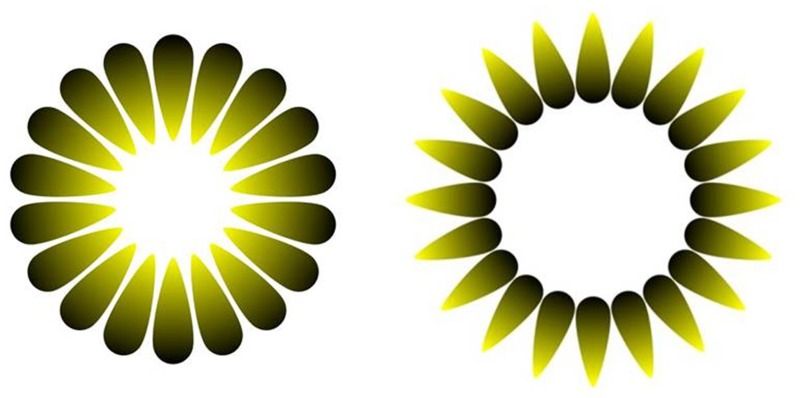
The brightness illusion (left) and its control image (right).

However, not every study has found atypical illusion perception in individuals with ASD (Manning, Morgan, Allen, & Pellicano, 2017; Ropar & Mitchell, 1999, 2001) and it seems likely that not every optical illusion may be affected (e.g., the Muller-Lyer illusion; Happé, 1996). Hence, it is relevant to document the occurrence and types of atypical perception in order to understand, at the empirical and theoretical levels, the whole constellation of cognitive changes that can occur in individuals with ASD. Specifically, charting what types of illusory phenomena are absent and which are preserved would contribute to identify perceptual and cognitive mechanisms that may be involved in the development of autistic features. For example, Maule, Stanworth, Pellicano, and Franklin (2016) have observed that color adaptation seems to function typically in adults with ASD (in contrast to reduced aftereffects for faces or numerosity; e.g., Ewing, Pellicano, & Rhodes, 2013; Turi et al., 2018). They concluded that, because color adaptation effects occur very early in the stream of visual processing (in part already in the retina), these effects may be less subject to differences in the influence of top-down expectations.

The previous studies included mainly children and adolescents and control groups matched in age. However, younger children are in general less prone to optical illusions than adults, and there appears to be a developmental progression with age in the strength of illusory perceptions (e.g., Kovács, 2000; Nayar, Franchak, Adolph, & Kiorpes, 2015). Hence, it is relevant to investigate individuals with ASD in other, older, age-groups.

In addition, studies of optical illusions in people with ASD (but also, in general, in individuals with typical development) have been based on introspective reports about size or shape judgments. Yet, it is also possible to study responses to illusions with behavioral responses (e.g., adjusting grip in the Ebbinghaus illusion) or with psychophysiological methods that measure responses that cannot be voluntarily controlled, such as the pupillary response. Indeed, in our laboratory, we found a constrictive response of the eye pupil to illusory brightness in individuals with typical functioning (Laeng & Endestad, 2012; Zavagno, Tommasi, & Laeng, 2017), as if the eye reacted (or prepared to react) to a physical increase in light emission or reflectance. Most relevant, we compared pupil responses to the illusory brightness display with an isoluminant control image, corresponding to 180° rotations of all the gradients that induce the brightness illusion (as illustrated in Figure 1, right), that does not engender the same experience of glare in the center of the image (in fact, it tends to yield the impression of a darker and duller region compared to original illusion’s central hole as well as the surrounding background). Since the constrictions in pupillary size when viewing the brightness illusion or its control image cannot be ascribed to changes in physical light energy or visual contrast, these findings strongly reveal the influence of perceptual hypotheses at a very early level of sensory processing (Laeng & Endestad, 2012).

This study explores the perception of brightness illusions in adults with ASD and a control group of adults with typical development. An infrared eye-tracker was used to measure the pupil diameters of the participants while they observed the illusion of brightness and a control image. If the group with ASD showed a weakened or absent illusory perception of brightness and related physiological changes, this would support the assumption that people with ASD tend to have a more veridical representation of the visual input (Pellicano & Burr, 2012). Consistent with the expected change in physiological responses, individuals with ASD should be less prone than controls to experience and report the brightness illusion.

## Methods

### Participants

Participants in the ASD group (*N* = 16) were recruited through *Autismeforeningen i Norge* (Norwegian Autism Association). All had been diagnosed with the ICD-10 (World Health Organization, 1992) by experienced clinicians. Five individuals with ASD were excluded from all analysis, since they were not able to keep fixation within a region of interest (ROI) of 3° of visual angle, centered on the fixation cross and overlapping the white central region within all patterns, for at least 90% of the time. Hence, 11 individuals with ASD were included in the analyses (8 men, mean age = 37.8 years, *SE* = 4.01; range = 23–65). The control group consisted of 24 individuals (10 men, mean age = 29.9 years, *SE* = 2.4; range = 23–66); the two groups did not differ significantly by age; *F*(1, 33) = 3.3, *p* = .08. All participants had self-reported correct or corrected-to-normal vision (by use of glasses or contact lenses). No participants had other neurological or psychological conditions. The final ASD group (*N* = 11) had an average percent dwell time around fixation = 95.5%, whereas the Control group maintained gaze within the ROI for a 96.9% of the time. The two groups did not differ in average pupil size at baseline (ASD = 11.3; *SE* = 0.5; Controls = 12.3; *SE* = 0.4). Eleven participants with ASD and 18 of the participants in the control group completed a test of fluid intelligence at the end of the session: the Standard Progressive Matrices by J. C. Raven (©Oxford Psychologist Press Ltd). The ASD group had a lower average score (mean = 36.5; *SE* = 3.1) than the Control group (mean = 49.1; *SE* = 1.4); *F*(1, 33) = 17.7, *p* = .0003. Written informed consent was obtained from all participants, and the project was approved by The Regional Committee for Medical and Health Research Ethics (REK Sør-Øst; approval number 2014/1192).

### Stimuli, Apparatus, and Procedure

The stimuli consisted of a version of the Asahi brightness illusion (Kitaoka, 2005), since earlier studies have found this to be a powerful illusory stimulus, as documented by the profile of perceptual responses or psychophysics as well as the physiological response of pupillary constriction (see Laeng & Endestad, 2012). As done in the previous investigations, responses to the brightness illusion were compared with those to a control picture (Figure 1) in which the illusory-inducing gradients diverge so as to disrupt, in fact reverse, the brightness illusory effect so that the central area appears relatively darker than the background in the control stimulus. In the pupillometry experiment, the brightness illusion and its darker control were shown in isolation for 4 s, and three colored patterns (yellow, purple, or green) were presented during the test, with size varying between 6° and 10° of visual angle, resulting in a total of six pseudorandomized trials intermixed to filler images of other geometrical patterns (e.g., checkerboard-like patterns). Participants were instructed to maintain fixation on the central cross throughout all trials and did not give any explicit responses to the stimuli.

The participants also completed a task where the three illusory patterns were shown pairwise and side by side (twice by positioning the “bright” pattern to the left and to the right of the “dark in each trial).” The participants reported verbally which of the two patterns looked brighter in its center.

SMI (SensoMotoric Instruments, Berlin, Germany) software (iView 3.2® *Experiment Center*) was used for presenting all experimental stimuli on a Dell P2213 VGA LCD 18.5′′ monitor with display resolution set to 1,680 × 1,050 pixels. Participants seated with their eyes 55 cm from the screen and they responded verbally to the experimenter who registered the responses in the computer.

In the pupillometry experiment with the bright illusion, after a simple 4-point calibration procedure, the eye-tracker sampled eye positions at a rate of 60 Hz using a SMI RED500 remote eye-tracking device by SensoMotoric Instruments (SMI, Germany). Right before the presentation of each pattern stimulus, isoluminant blank images appeared for 1,000 ms. Pupil responses (in mm) to each baseline were subtracted from the corresponding pupil response to a pattern so as to obtain the main dependent variable of “pupillary change.” During the task, participants maintained fixation on a little dot at the center of each pattern without making any verbal or manual response.

## Results

**Figure 2.**
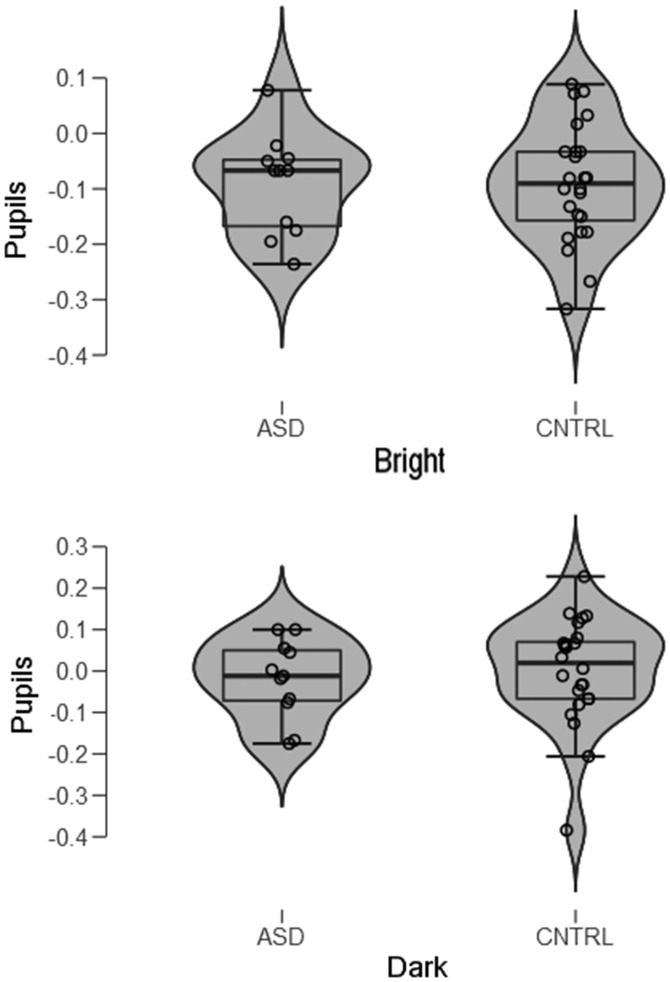
“Violin plots” of mean pupillary changes in millimeters to the (bright) illusion (top panel) and the (dark) control pattern (bottom panel) in the ASD group and the CNTRL group. The plots show the density of the data with markers for the median of the data and the interquartile range. Overlaid on this box plot is a kernel density estimation. ASD = autism spectrum disorder; CNTRL = Control.

All measures of pupil size in a same individual and to the bright and dark stimuli, from onset until the offset of the stimulus (4,000 ms), were averaged to obtain a mean pupillary change. These were first analyzed in a repeated-measure analysis of variance (ANOVA) with Group (ASD vs. Controls) and Illusion (bright vs. dark) as the between-subject and within-subject factors. Contrary to the prediction that the effect of brightness illusions on pupil sizes would be weaker or absent in the ASD group than the control group, there was no indication of a differential pupillary change when looking at the illusory bright versus dark stimuli. As expected and shown in Figure 2, pupils did constrict to the bright illusions, *F*(1, 33) = 17.8, *p* = .0002. This finding replicates the presence of a pupillary reduction to bright illusions previously reported by Laeng and Endestad (2012).

**Figure 3.**
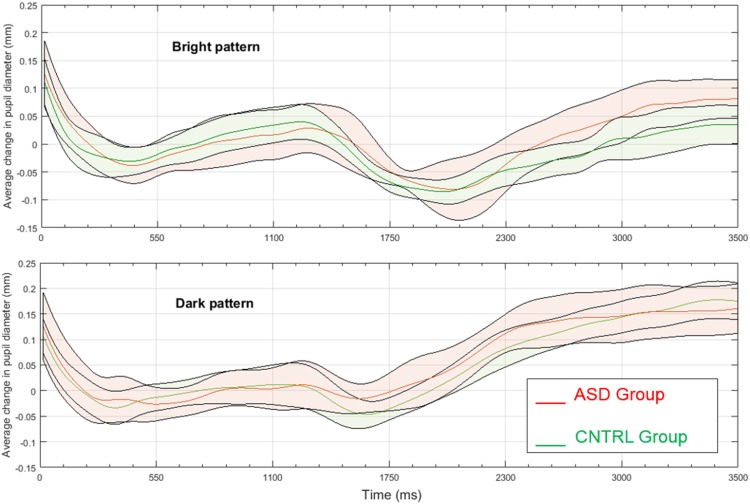
Mean pupillary change in millimeters to the “bright” pattern (top panel) and the “dark,” control pattern (bottom panel) in the ASD group (red lines and shading) and the Control group (green lines and shading). The shaded stripes indicate 95% confidence intervals around the mean. ASD = autism spectrum disorder; CNTRL = Control.

There were, however, neither a main effect of Group, *F*(1, 33) = 0.87, *p* = .77, nor an interactive effect of Illusion and Group, *F*(1, 33) = 0.24, *p* = .63. The effect sizes of the responses of the ASD group and Control group to the “bright” or the “dark” patterns appeared to be similar (bright: Cohen’s *d* = 0.0; dark: Cohen’s *d* = 0.179). When exploring the illusory effects in the two groups with separate ANOVAs, there was a strong effect size of the Illusion in both the Control group (Cohen’s *d* = 0.813; Lambda = 17.8; Power = .99) and the ASD group (Cohen’s *d* = 0.779; Lambda = 7.5; Power = .70). Figure 3 illustrates the pupillary change evolution in each group for the “bright” and “dark” patterns; as the shaded stripes (i.e., the 95% confidence intervals around the mean) indicate, the graphs reveal a substantial overlap in pupillary changes over time in the two groups.

In addition, we analyzed the present results with a Bayesian repeated-measures ANOVA using the statistical software *JASP* (https://jasp-stats.org/). This analysis had Group (ASD vs. Controls) and Illusion (bright vs. dark) as the between-subject and within-subject factors. We used as prior a value of 0.2, which is the default for Bayesian repeated-measures ANOVA in *JASP*. We found that the Group’s BF_10_ = 0.363 and the inclusion Bayes Factor (based on matched models) for the Group × Illusion interaction is 0.391, indicating that the present results are 2.56 more likely under H0 than under H1. However, the Illusion factor had a BF_10_ = 653.4, indicating strong evidence for a pupillary difference when viewing stimuli like the ones in Figure 1.

Consistent with their pupil responses, both groups made similar judgments of relative brightness, as the (“bright”) pattern was judged as “brighter” than the (“dark”) control in its center by both the individuals with ASD (Mean % choices = 81.7 %; *SE* = 8.1) and the controls (Mean % choices = 86.9; *SE* = 5.9), *F*(1, 28) = 0.24, *p* = .63.

A series of regression analyses failed to reveal a predictive role of either participants’ age on either mean % “brighter” choices (*r* = .02, *p* = .93) or the mean pupil changes to the bright illusion (*r* = .09, *p* = .90). Similarly, scores in the fluid intelligence test (Raven Matrices) failed to predict mean pupil changes to the bright illusion for either ASD (*r* = .003, *p* = .99) or Control participants (*r* = .11, *p* = .68), as well as the participants’ mean % “brighter” choices (*r* = .08, *p* = .69).

## Discussion

This study is the first exploration of pupillary responses to illusory brightness in individuals with ASD. Based on recent accounts of “veridical” perception in this group (e.g., Mottron et al., 2013; Pellicano & Burr, 2012), it was hypothesized that the ASD group would differ from the control group by showing an absent or reduced constriction of the pupil to illusory light as well as a reduced proportion of reports of illusory effects. This prediction was based on recent accounts of increased reliance on sensory input or reduced reliance on perceptual hypotheses (or “hypo-priors” or “aberrant precision” of predictions; Lawson et al., 2014; Pellicano & Burr, 2012) in individuals with ASD compared to individuals with typical development.

The present results did not reveal a difference in either perceptual reports or physiological adjustments to the above patterns. Therefore, this study does not support the hypothesis that the optical illusion of enhanced brightness or glare differs in the perceptual processing of individuals with ASD. Such a negative conclusion is consistent with other failures to replicate a veridical perception of illusions (Manning et al., 2017; Ropar & Mitchell, 1999, 2001). One should also consider that the physiological evidence collected here would seem immune to effects of task demands (Rosenthal & Rubin, 1978), since the pupil response cannot be controlled at will (Laeng & Sulutvedt, 2014) and constitute a “honest signal” related to the experience, whereas responses in the typical tasks can be controlled voluntarily (e.g., either responding verbally or when performing adjustments of the perceptual elements, like in Ropar & Mitchell, 1999, 2001). Thus, based on the present findings, it seems instead that atypical processing related to visual illusions does not constitute a universal aspect of visual processing in individuals with ASD and it is not a necessary element for the development of other difficulties related to ASD (cf., Behrmann et al., 2006). In fact, any single formulation of cognitive changes in people with ASD may fail to capture the heterogeneity and multifactorial causality below the phenomena observed (Happé, Ronald, & Plomin, 2006).

Interestingly, a recent study by Blaser, Eglington, Carter, and Kaldy (2014) examined pupil responses of two groups of toddlers, one with ASD and one with typical development, during visual search and found that the ASD group had larger dilations during this attentional task than the control group. The authors suggest that the increased pupil size of the ASD group was a sign of increased attentional focus, which led this group to perform better than children with typical development. However, a similar explanation is unlikely in this study, since passive viewing of visual patterns is not as demanding as visual search, and the present findings indicate equal constrictions to the brighter illusions and no sign of an opposite, dilation, pupil response in the ASD group. In addition, recently, Lawson, Mathys, and Rees (2017) showed that adults with ASD had a reduced dilation response to surprising outcomes when learning about probabilistic relationships. They interpreted this finding as resulting from a tendency to overestimate the “volatility” of the sensory environment. In the present context, as suggested in Zavagno et al. (2017), automatic constrictions to subjective brightness suggest the presence of predictions of forthcoming “glare” that prepare the visual system to a probable increase in light energy and the pupil adjusts its size as a “protective response” to anticipated dazzle. These fast pupillary constrictions to probable blinding sunlight could be evolutionarily selected to reduce its threat to survival and therefore may not result from an individual’s learning about the volatility of the sensory environment. Finally, Turi, Burr, and Binda (2018) showed that scores in the Autism Spectrum Quotient (Baron-Cohen et al., 2001) in a group of adults with typical development were related to pupillary changes when reporting the direction of motion of a bistable rotating object. These changes were interpreted as indicating adjustments of the focus of visual attention (as in Binda, Pereverzeva, & Murray, 2014; Mathôt & van der Stigchel, 2015) to either the brighter or darker surfaces of the object. Turi et al.’s results show that traits typical of ASD relate to different ways of focusing attention and, in turn, affect pupil size. However, Turi et al. did not test participants with ASD. Nevertheless, on the basis of their study, we surmise that similar deployments of attention took place in our ASD group and in the adults with typical development when viewing the brightness illusions. In general, it seems likely that the glare illusion effect actually depends on “predictive processes” of a different kind than those that affect other illusory effects (Laeng & Endestad, 2012; Zavagno et al., 2017).

We need to stress that there are several limitations in this study. First of all, it is based on a small sample of participants with ASD (*N* = 11) compared to other studies (e.g., *N* = 25 in Happé, 1996). However, the study of Ropar and Mitchell (1999, 2001) was based on 36 and 30 individuals with ASD, and these studies also failed to observe differences. In fact, in this study, we found no hints that the pupil responses of the participants with ASD differed on average or in their variability to those of the controls, as indicated by the comparable effect sizes of physiological response of the pupils and the individuals’ distribution of pupil change. The Bayesian analysis leaned toward only “weak” if not inconclusive evidence (Dienes, 2014, or “anecdotal,” cf. Jeffreys, 1961) for the null hypothesis, since it found the present results to be 2.6 times more likely under the null hypothesis than what predicted by a “veridical perception” account of ASD. We also note that this study investigated adults instead of children, and the perception of illusion is more common in adults than in younger children (cf. Doherty, Campbell, Tsuji, & Phillips, 2010). Hence, one possible explanation for the difference in results from Happé (1996) may be that results with children with ASD reflect a delay rather than a deviance from the typical developmental course. However, the studies by Ropar and Mitchell (1999, 2001) and Manning et al. (2017), which also did not find significant differences between ASD and control groups, had younger participants than this study (mean age around 13 and 10 years, range = 7–18 and 6–14, respectively), and comparable to Happé (1996; mean age 13, range 8–16). An adult group in Ropar and Mitchell (1999) did not differ systematically from the other groups. Moreover, we note that our groups were not optimally matched with regard to fluid intelligence and genders’ proportions, the former being higher in the control group and the proportion of females lower in the ASD group. However, we do not assume illusory perception to be influenced by differences in IQ in adults. In younger subjects, it is more likely that a lower susceptibility might reflect more immature perceptual function, but this would constitute an influence that would work against the present results. Similarly, there is no known gender difference in susceptibility to brightness illusions. Finally, this study did not include a measure of severity of symptoms in the ASD group or of autistic traits in the control group. However, given the small group of individuals with ASD that we were able to recruit in this study, this information would not have been likely to reveal significant effects. Future studies, with larger and better matched samples, should further address more detailed relationships between symptoms of ASD and the perception of illusory objects.
